# Genome-Wide Identification and Expression Analysis of Dof Transcription Factors in Lotus (*Nelumbo nucifera* Gaertn.)

**DOI:** 10.3390/plants11152057

**Published:** 2022-08-06

**Authors:** Xiaohan Cao, Wenyang Wan, Huimin Mao, Dandan Yin, Xianhui Deng, Huan Yan, Liping Ren

**Affiliations:** 1 Key Laboratory of Horticultural Plant Biology, Biological and Food Engineering School, Fuyang Normal University, Fuyang 236037, China; 2 Fuyang Academy of Agricultural Sciences, Fuyang 236065, China

**Keywords:** *Nelumbo nucifera*, Dof transcription factor, salinity stress

## Abstract

Lotus (*Nelumbo nucifera* Gaertn.) is a traditional Chinese aquatic flower with high ornamental and economic value, but water salinity seriously affects lotus cultivation and distribution. The Dof transcription factors (TFs) play a crucial function in the regulatory network of growth and defense in plants. However, no systematic investigations of the Dof TFs in lotus have been performed. In this study, comprehensive searches of the lotus genome yielded 29 potential *NnDofs*. We carried out a series of standardized analyses, which include physical properties, multiple sequence alignment, phylogenetic analysis, gene structure, motif composition, cis-acting element prediction, chromosome distribution, and synteny analysis. The results showed that segment duplication probably caused the *NnDofs* gene family expansion. The potential functions of *NnDofs* in lotus development and stress conditions are speculated by promoter analysis. Furthermore, a complete expression investigation of *NnDofs* utilizing an RNA-seq atlas and quantitative real-time polymerase chain reaction (qRT-PCR) was performed. The majority of the *NnDofs* exhibit tissue-specific expression patterns, and many genes have been identified as being extremely sensitive to salt stressors. Overall, this study is the first to report a genome-wide assessment of the Dof family in lotus, and the findings offer vital insights for prospective functional studies on lotus salinity stress.

## 1. Introduction

Soil salinization is a serious and growing global problem [1], and salinized areas are growing at a rate of 10% per year. Soil salinity is a severe abiotic stress that affects plant seed germination, growth and development, and reproductive development by causing oxidative stress, ionic toxicity, osmotic stress, and metabolic disturbances in plants [2,3]. Several investigations on several plant species have highlighted the complicated and crucial role of transcription factors in abiotic stress reduction [4]. The dynamic coordination of salt stress-responsive transcription factors in the interaction pathway, as well as their unique integration into the stress adaption cellular network, will serve as a stepping stone for plant tolerance to environmental stresses [5].

Transcription factors (TFs), also called trans-acting factors, interact with cis-acting elements in a particular genetic promoter region to regulate gene transcription and ensure target gene expression at a specific time, place, and intensity [6,7]. Typical transcription factors contain functional regions such as the DNA binding domain, nuclear localization signal region, oligomerization site, and transcription activation domain [8,9]. Transcription factors widely regulate plant growth and development and deeply engage in biotic and abiotic stress responses.

Studies have shown that Dof (DNA binding with one finger) proteins appear to be unique to plants. The first Dof transcription factor identified was *ZmDof* from maize [10]. With the development of bioinformatics technology, more Dof proteins have been found in the genomes of different plant species, such as *Solanum lycopersicum* [11], *Manihot esculenta* [12], *Prunus persica* [13], and *Capsicum annuum* L. [14]. Dof TFs usually consist of 200–400 amino acids with highly conserved DNA-binding domains at their N-termini, transcriptional regulatory domains at their C-termini, and nuclear localization signals [10,15]. The highly conserved domain consisting of 50–52 amino acids at the N-terminus, containing a C2-C2 zinc finger domain is composed of CX2CX21CX2C. Different transcriptional regulatory domains at the C-terminus indicate the diversity of Dof protein functions [16]. The *Dof* family was divided into seven sub-populations by Yanagisawa [17]. Several researchers collected Arabidopsis and rice *Dof* genes and classified them into four subfamilies: Aa, Bb, Cc, and Dd [18]. The 116 *Dof* genes from seven species were more comprehensively classified by Moreno-Risueno into seven subgroups, A–G [19]. *Dof* proteins play multiple roles in different biological processes, including growth and development [20,21], flowering regulation [22,23], carbon and nitrogen metabolism [24], hormone response [25], and abiotic stress [26,27] in various plant species. Overexpression of *OBP4* in Arabidopsis promotes cell proliferation in the differentiation zone and induces callus formation [28]. *ZmDof3* controls starch accumulation and aleurone development in maize endosperm by binding to the *Dof* core element promoters of *Du1* and *Su2* [29]. In addition, *ZmDof36* is important in regulating starch synthesis. Its overexpression can increase starch content and reduce soluble sugars and reduce sugars [30]. Cycling Dof factor 2 (*CDF2*) leads to photoperiod-insensitivity and delayed flowering in Arabidopsis by reducing CO mRNA levels [31].

RNA-Seq data showed that most *TaDof* genes respond to heat and PEG-induced drought stress in wheat [26]. Overexpression of *GhDof1* could notably enhance tolerance to salt and cold stresses by increasing proline content during the seedling stage [32]. Some *ClDof* genes showed significantly different expressions under salt stress, suggesting that they may contribute to salt stress adaptation in watermelon [33]. *OsDof15* coordinates the regulation of salt and ethylene, inhibiting primary root growth by affecting cell proliferation in the root apical meristem [34]. *SlDof22* can be combined with the promoter of the *SlSOS1* gene, and inhibiting *SlDof22* by significantly downregulating the *SlSOS1* gene leads to reduced tolerance to salt stress [35].

Sacred lotus (*Nelumbo nucifera* Gaertn.), which has been grown in the Far East for 5000–7000 years, is a large aquatic plant with significant ecological, scenic, and economic value [36,37]. Lotus cultivars are categorized depending on their usage and morphological characteristics: rhizome lotus, seed lotus, and ornamental lotus [38]. Lotus has high ornamental value throughout the growing period, with large flowers, various petal types, gorgeous color, green leaves, tall and straight habit, and remains attractive even during the dry leaf period. Because of its ornamental value, lotus is considered a theme plant in waterscape garden layouts. In addition, lotus has high economic value and medicinal value [39]. Lotus tea is traditionally used to clear away “summer heat”, that is, relieve symptoms of heat injury, and lotus seed is rich in phospholipids, alkaloids, and flavonoids, which in Chinese traditional medicine are used to clear the heart, nourish the mind, and tonify the spleen and kidney [40].

Although salinity stress causes certain harm to the growth and development of lotus, there are few reports on the salt tolerance of ornamental lotus. Furthermore, Dof TFs have been found to be resistant to salt stress in many different plant species, but the *Dof* gene has not been identified in lotus. In this study, we identified and characterized 29 *Dof* family genes in lotus. They were unevenly distributed on the seven chromosomes and divided into six groups based on phylogenetic analysis. Its physicochemical properties, gene structure, conserved motifs, and cis-acting elements upstream of the gene were also analyzed. Tissue-specific expression of *NnDofs* and gene response to salt treatment were investigated using RNA-seq data and qRT-PCR. We predicted possible interacting proteins and regulatory networks of *NnDofs* related to these stress responses. The results provide a reference for further functional research of lotus *Dof TFs*, and they can be used as genetic resources to make lotus and other crops more tolerant of salt through molecular genetic breeding.

## 2. Results

### 2.1. Identification and Physiochemical Characteristics of NnDofs

Through the search of the lotus genome by HMM with the Dof domain (PF02701), 29 *NnDof* family members were recognized after further validation in the CDD and Pfam databases. According to the distribution of *Dof* genes on chromosomes, *NnDofs* are named *NnDof1* to *NnDof29*. We investigated the physical and chemical properties of the *NnDof* members. Analysis of NnDof proteins showed amino acid lengths ranging from 105 to 508 aa, molecular weights ranging from 12.1 to 55.4 kDa, and isoelectric points ranging from 5.67 (NnDof13) to 9.96 (NnDof29). The analysis results of the instability index showed that the *NnDof5* value was lower than 40, which indicated that the rest of the *NnDofs* were probably unstable proteins. The aliphatic indexes of NnDof proteins were all between 41.81 and 73.51. GRAVY values for all NnDof proteins ranged from −1.161 to −0.341, indicating that all NnDof members are hydrophilic proteins. The subcellular localization prediction findings indicated 17 NnDof proteins in the nucleus and the remainder are extracellular. Detailed results are listed in Table S1.

### 2.2. Phylogenetic Analysis and Classification of NnDofs

**Figure 1:**
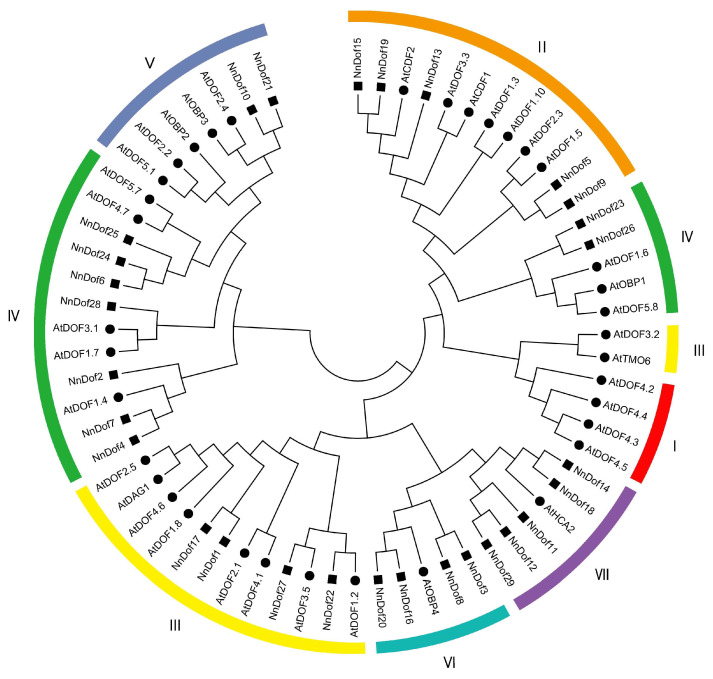
Phylogenetic tree of Dof transcription factors in *N. nucifera* and *A. thaliana*. The phylogenetic tree was created using MEGA 6.0, with the neighbor-joining method and bootstrap value set at 1000. Black circles represent the 36 AtDof proteins, and the 29 NnDof proteins are marked with black squares. The resulting phylogenetic tree was clustered into seven main groups, labeled I, II, III, IV, V, VI, and VII.

To fully comprehend the evolutionary relationship, a phylogenetic tree was constructed with the 29 identified *NnDofs* and the 36 reported *AtDofs* of Arabidopsis (Figure 1). The results indicated that 65 Dof proteins were grouped into seven major groups, with 29 NnDof proteins in each of the following categories: II, III, IV, V, VI, and VII. Group VI had nine NnDof proteins, accounting for 31.03 percent of the total number of *NnDofs*, while group V had only two, accounting for 6.90 percent. Groups II, III, VI, and VII each have five NnDof proteins.

**Figure 2:**
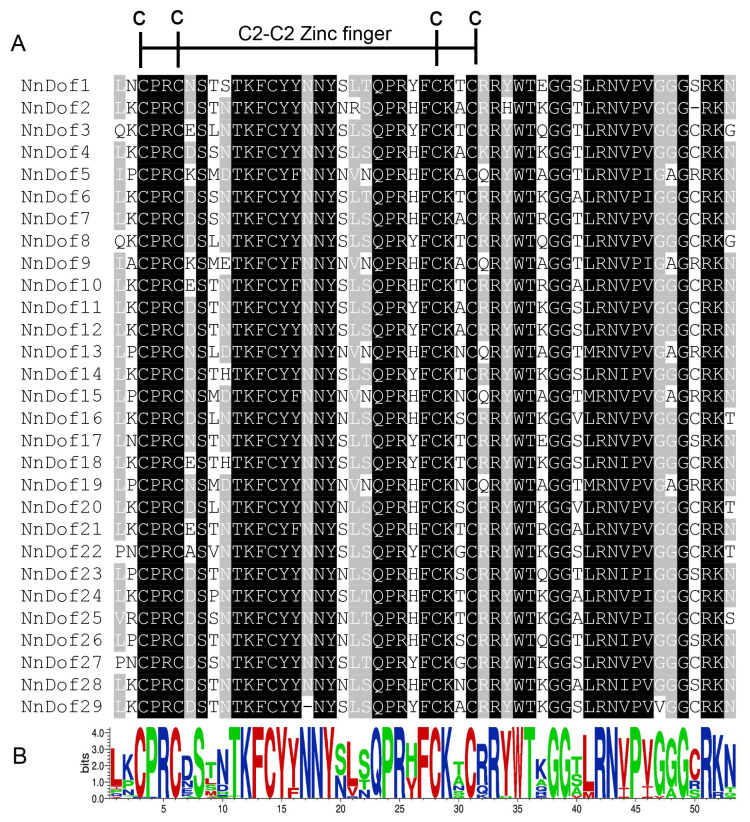
Dof domain sequence alignment of NnDof proteins. (**A**) Extraction of conserved domains of Dof proteins (black region—amino acid homology 100%; grey area—amino acid homology 75%); (**B**) the conserved domains shown by WebLogo.

Multiple sequence alignment analysis of the amino acids of the Dof structural domain of 29 lotus flowers was performed to investigate the sequence characteristics of the NnDof proteins. It was found that the NnDof proteins’ structural domain sequences were highly conserved and all contained the typical CX2CX21CX2C motif, where one Zn^2+^ can covalently combine with four Cys residues (Figure 2).

### 2.3. Gene Structure and Conserved Motifs of NnDofs

**Figure 3:**
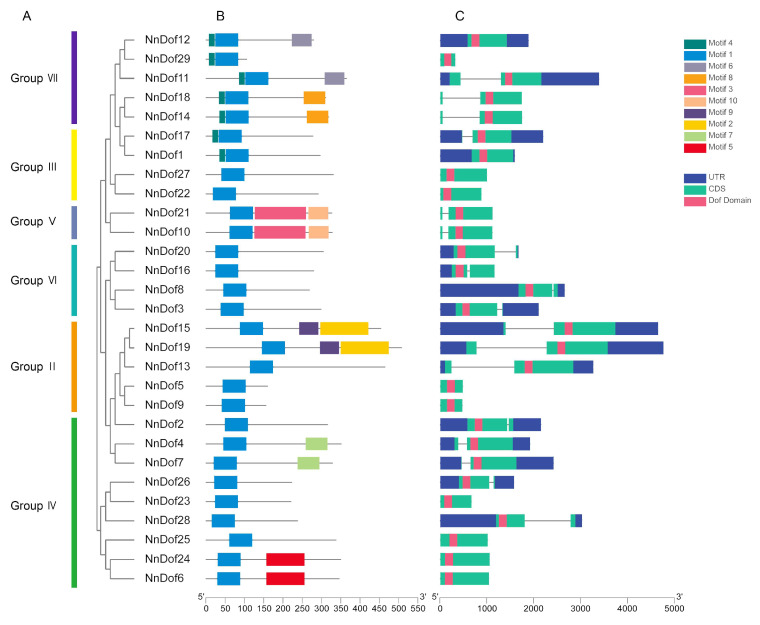
The phylogenetic relationships, exon–intron structures, and motif compositions of lotus Dof proteins. (**A**) The phylogenetic tree was constructed based on *NnDofs*, constructed using MEGA7 with a bootstrap of 1000 by the neighbor-joining method. (**B**) Untranslated regions (UTRs), exons, introns, and Dof domain are represented by blue, green, black lines, and pink, respectively. (**C**) A total of 10 motifs are denoted by different colored boxes.

We investigated the conserved motifs of the genes using MEME to evaluate the diversity and conservation among all 29 *NnDofs* genes. Ten different motifs were predicted and they ranged in size from 16 to 134 amino acids (Figure 3B). The conserved structural domain of Dof consists of Motif 1, and Motif 1 corresponds to the CX2CX21CX2C single zinc finger structure in the Dof structural domain, which is a highly homologous core region of the *Dof* family. NnDofs proteins associated on evolutionary branches of the phylogenetic tree have identical or comparable motif structures. Some motifs, such as Motif 2, Motif 3, Motif 5, and Motif 8, are only found in certain subgroups and may be connected to different functions.

Introns and exons constitute genes, and their numbers and distribution patterns serve as an evolutionary indicator for a gene family. Thus, we completed a comparison of the intron-exon structures of each *NnDofs* (Figure 3C). In general, the number of introns per *NnDof* gene varied very little, ranging from 0 to 1. A total of 11 *NnDof* gene members had no introns (37.9%) and 18 members had only one intron (62.1%). The exon-intron structure pattern of the *NnDofs* gene is similar to that of Arabidopsis, rice, and tomato. Most introns are usually located upstream of the *Dof* structural domain, and only seven genes (*NnDof2*, *NnDof3*, *NnDof8*, *NnDof16*, *NnDof20*, *NnDof26*, and *NnDof28*) have introns located downstream of the *Dof* structural domain. Most of the genes in the same groups showed similar exon and intron patterns. For example, most *Dof* genes in group III do not contain introns. In contrast, groups II, V, VI, and VII almost all contain one intron.

### 2.4. Promoter Analysis of NnDof Genes

**Figure 4:**
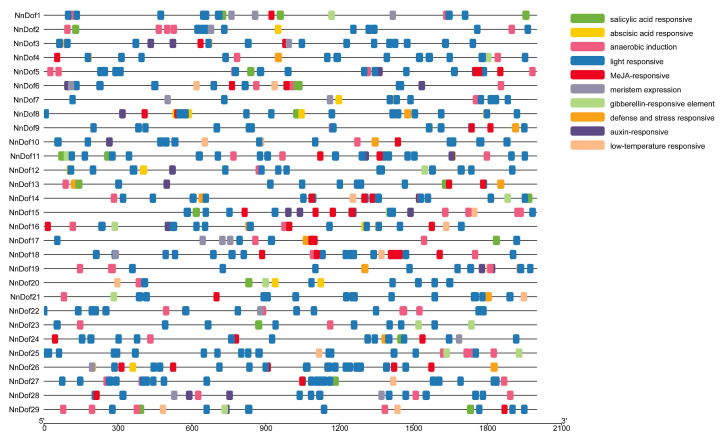
The prediction of a cis-acting element in the 2000 bp promoter upstream of the *NnDofs*. The main cis-acting elements are marked in the upper right corner.

The prediction of cis-regulatory elements in *NnDof* gene promoters can help us learn more about how gene family members regulate transcription (Figure 4). All 29 *NnDofs* have TATA-box and CAAT-box. It was discovered that light-responsive elements (ATTAAT, CACGTG, and GGTTAA) and meristem expression-related elements (GCCACT) are extensively distributed in the promoter area of the *NnDofs*. There were various elements implicated in abiotic stress responsiveness, including low-temperature responsive elements (CCGAAA), salicylic acid responsive elements (CCATCTTTTT and TCAGAAGAGG), anaerobic induction response elements (AAACCA), and other defense and stress responsive elements (ATTCTCTAAC). Abscisic acid-responsive elements (ABREs) such as (ACGTG, CACGTG, TACGGTC, and TACGTG), MeJA-responsive motifs such as (TGACG and CGTCA), gibberellin-responsive motifs such as (TCTGTTG and CCTTTTG), and auxin-responsive elements such as (GGTCCAT and AACGAC) were widely found. These results reveal that *NnDofs* are not only regulated by stress responses, but may also be involved in plant growth and development.

### 2.5. Interaction Network Analysis of NnDofs

**Figure 5:**
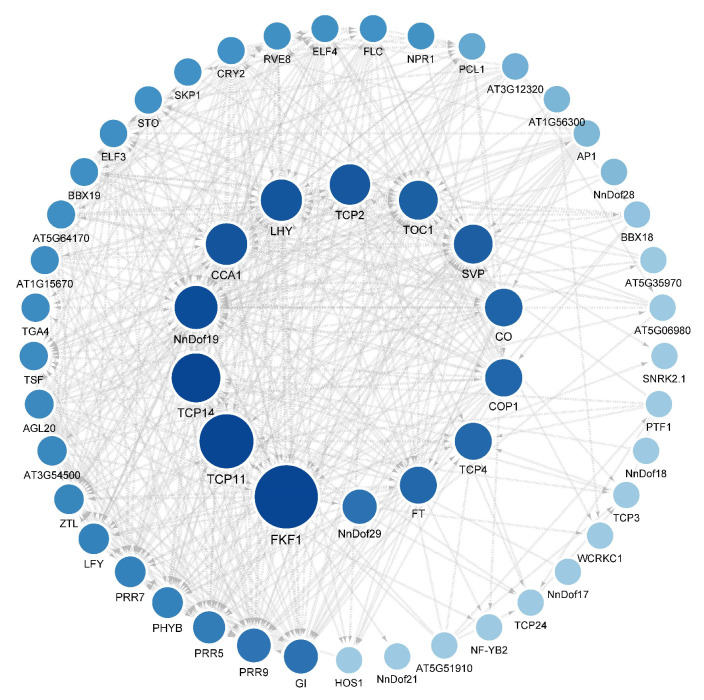
Protein interaction network of NnDofs protein with other Arabidopsis proteins predicted using the STRING-DB. The minimum required interaction score is a moderate confidence level of 0.400. Organism set: Arabidopsis. These lines reflect the interaction of Nndofs homologs with other proteins. The darkness of the circle color indicates the strength of the data support.

The protein interaction network relationship of *Dof* genes was predicted with orthologous genes in STRING to analyze the potential biological function and regulatory network of *NnDofs*. The results showed that eight *NnDofs* were orthologous to *Arabidopsis thaliana*. The protein–protein interaction (PPI) network with obvious interaction relationship for all *Dofs* included 55 nodes and 461 edges (Figure 5). In addition, the top five pairs with the highest combined score among all PPI networks of NnDofs proteins are NnDof28-TGA4 (0.927), NnDof19-PRR9 (0.893), NnDof29-TCP14 (0.893), NnDof19-STO (0.864), and NnDof29-TGA4 (0.834). Further functional enrichment was performed on the proteins of the PPI network. Most of the proteins interacting with *NnDofs* were related to circadian rhythm-controlled flowering regulation, while others were related to defense response to bacterium (TGA4), growth and development (IQD11), and seed germination regulation (TCP14). The network analysis of these protein–protein interactions provides clues for studying the potential functions of *NnDofs*, and also lays the foundation for further study of candidate genes.

### 2.6. Chromosome Distribution, Gene Duplication and Synteny Analysis of NnDofs

**Figure 6:**
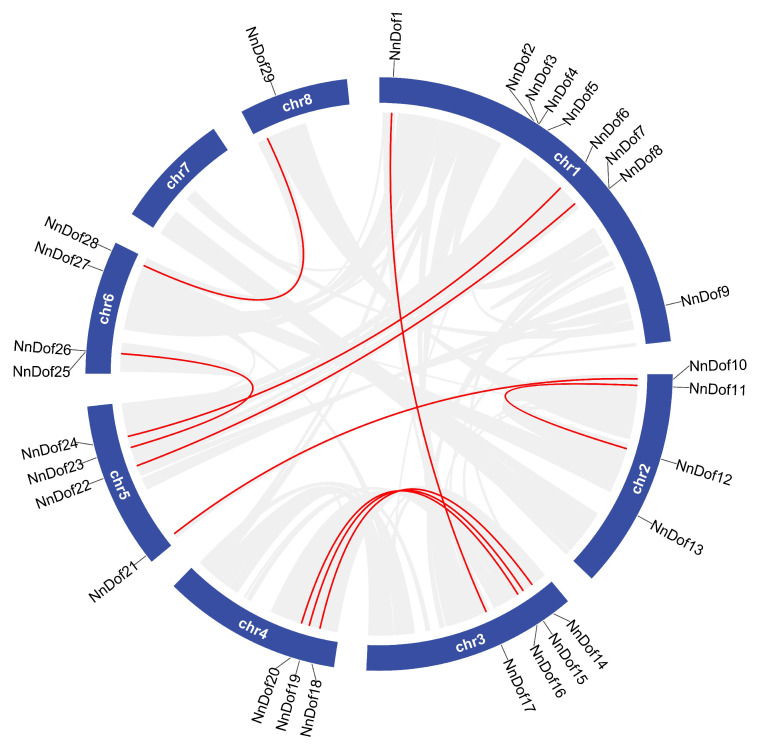
Chromosomal locations and segmental duplication events of NnDofs. Red lines represent segmental duplication events.

We investigated the evolution and expansion mechanisms of *NnDofs* genes by assessing their chromosomal location and gene duplication events. The number distribution of *NnDofs* on chromosomes was heterogeneous, with 29 *NnDofs* located on seven of the eight chromosomes and nine *NnDofs* found on Chr 1, but no *NnDof* genes located on chr 7 (Figure 6). *NnDofs* are localized in the front end of chr3 and chr4 but are separated in chr1, chr2, and chr5. Both tandem and segmental duplications contribute to gene family expansion. We evaluated duplication events in *NnDofs* and highlighted 10 segmental duplications of *NnDofs* gene pairs with red lines. Based on the research published by Holub, two pairs of *NnDofs*, NnDof3-NnDof4 and NnDof25-NnDof26, were identified as tandem duplications.

**Figure 7:**
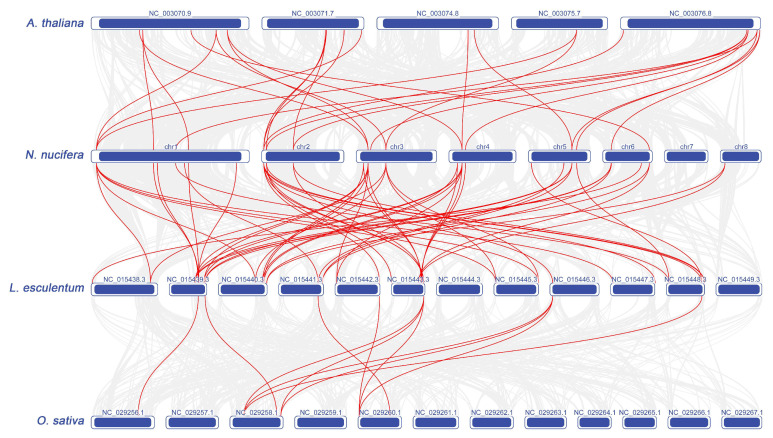
Synthetic analysis of *NnDofs* in lotus, rice, tomato, and Arabidopsis genomes. The red lines represent homologous gene pairs between two adjacent species.

We also conducted a synteny analysis of *Dof* genes across lotus, tomato, Arabidopsis, and rice (Figure 7). There are 26 orthologous pairs between lotus and Arabidopsis, and 53 orthologous pairs between lotus and tomato. Six *NnDofs* in lotus (*NnDof11*, *NnDof12*, *NnDof15*, *NnDof17*, *NnDof19*, and *NnDof23*) have two pairs of homologous genes in Arabidopsis, and two *NnDofs* (*NnDof1* and *NnDof10*) have three pairs of homologous genes in Arabidopsis thaliana. Analysis of the number of collinear homologous genes between lotus and tomato revealed that four *NnDofs* (*NnDof12*, *NnDof14*, *NnDof18*, and *NnDof28*) have two homologous genes in tomato, three *NnDofs* (*NnDof1* and *NnDof17*) have four homologous genes in tomato genes, and three *NnDofs* (*NnDof10*, *NnDof15*, and *NnDof19*) have five homologous genes in tomato. A total of 11 *SlDofs* genes showed syntenic relationships with those in rice.

### 2.7. Expression Patterns of NnDofs in Lotus

**Figure 8:**
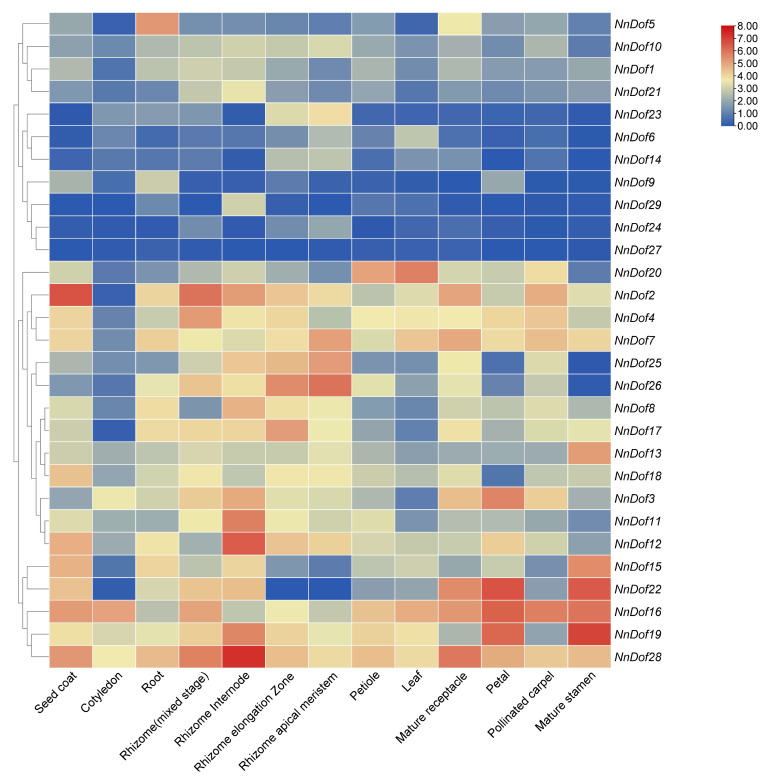
Expression pattern of *NnDofs* in different tissues of lotus. The expression pattern was generated based on FPKM plus 1 after log2 transformation and analyzed by heatmap hierarchical clustering.

RNA-seq data were used to discover how *NnDof* was expressed in different tissues of the lotus (Figure 8). We investigated the expression patterns of *NnDofs* since their distinct physiological roles are interrelated with their expression patterns in different tissues and at different developmental stages. The expression patterns of *Dof* genes in different tissues and organs may be grouped into three groups based on clustering results, and members of the same group have comparable expression profiles. In group 1, *NnDofs* expression was low in most tissues; in group 2, certain *NnDofs* were dominantly expressed in some tissues; and in group 3, *NnDofs* expression was high in most tissues. In general, genes have similar expression patterns. For example, some genes, such as NnDof19 and *NnDof28*, are highly expressed in all tissues, while *NnDof24* and *NnDof27* are lowly expressed in all tissues, and *NnDof2*, *NnDof16*, *NnDof22*, and *NnDof28* are predominantly expressed in the seed coat. Furthermore, 26 (90%) *NnDofs* were expressed in at least one tissue and developmental period (log_2_^(FPKM)^ > 0); six (21%) *NnDofs* were expressed in all samples tested, which suggests that these *NnDofs* act at multiple developmental stages; and only one (3%) *NnDof* was lowly expressed in all samples tested and may be specifically expressed in other tissues and developmental periods.

**Figure 9:**
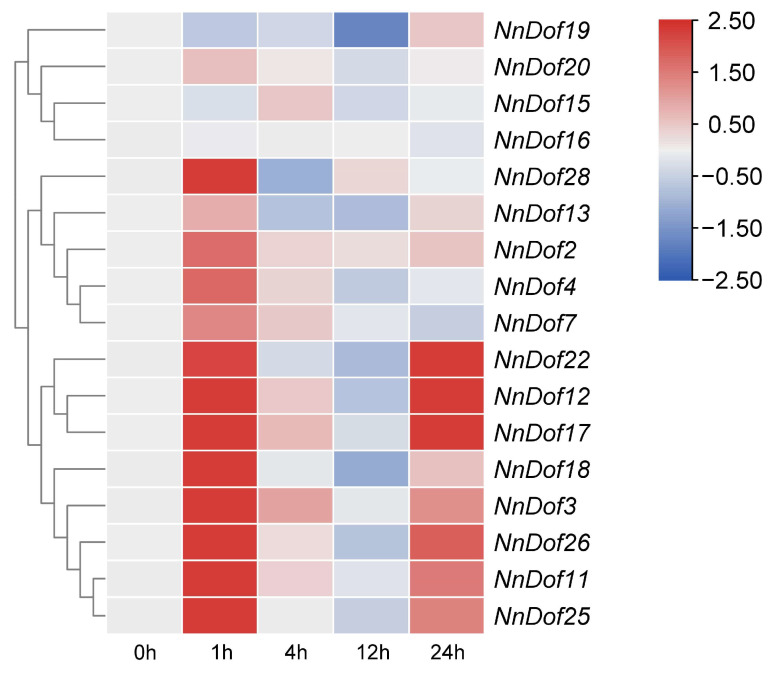
Expression profiles of *NnDofs* genes responding to salt stress. Blue indicates decreased expression levels and red indicates increased expression levels. Heatmaps were generated with TBtools software.

Whole plant leaves were used when extracting total RNA to prepare for the qRT-PCR analysis, which was validated to discover how the NnDofs genes were expressed under salt stress (Figure 9). Under salt stress, the expression of *NnDofs* was rapidly upregulated at 1 h, except for *NnDof15*, *NnDof16*, *NnDof19*, and *NnDof20*. Some *NnDofs* genes were downregulated at 4 h, 12 h, and 24 h, but *NnDof3*, *NnDof11*, *NnDof12*, *NnDof17*, *NnDof18*, *NnDof22*, *NnDof25*, and *NnDof26* were upregulated at 24 h.

## 3. Discussion

The ornamental lotus is a traditional Chinese flower and an important aquatic plant, which is loved and widely cultivated for its brilliant color, rich flower shape, and fragrant smell. It has been reported that common lotus species are sensitive to salt stress, and increasing soil salt concentration can significantly affect their growth and development processes, further affecting lotus cultivation and production. Therefore, mining and analyzing the genes associated with salt stress resistance in lotus is important to improve the resistance of lotus. *Dofs* have been reported to play an important role in salt stress resistance in rice and tomato. In this study, we performed a genome-wide search for *NnDofs* with a focus on salt stress resistance. This will help us learn more about how *Dof* genes work and how to improve the genetics of lotus.

### 3.1. Identification, Structural and Phylogenetic Analysis, and Evolutionary Characterization of NnDofs

Assessing the structure and function of transcription factors facilitates the analysis of species-specific gene regulatory networks [41]. *Dof* genes are plant-specific transcription factors with important roles in plant growth and development [17]. Other functions of most *Dof* genes remain to be explored. *Dof* genes have been identified in Arabidopsis (36), rice (30), maize (46), tomato (34), and chrysanthemum (20) species. A total of 29 *Dof* genes were identified in the lotus genome, which is close to the number of *Dof* genes in rice and tomato. Systematic classification has important implications for the analysis of *NnDofs*. Systematic classification has important implications for the analysis of *NnDofs*. The Arabidopsis Dof proteins were incorporated with NnDof proteins to construct a phylogenetic tree, and *NnDofs* were classified into six of seven subfamilies. Interestingly, none of the *NnDofs* were classified into group I, while *AtDOF4.2* and its close homolog *AtDOF4.4*, which are classified in group I, are associated with regulation related to the regulation of branching meristem seed coat formation in Arabidopsis [42]. In addition, overexpression and RNAi-mediated silencing of *AtDOF4.2* have opposite effects on the expression levels of flavonoid biosynthesis-related genes and flavonoid accumulation [43].

Gene structure and motif distribution can be used as supporting evidence for evolutionary relationships between species or genes [44,45], while generally, members of the same subfamily have similar exon/intron structure and number distribution [19]. However, the distribution of the number of introns in the taxonomic subgroups of lotus did not follow this pattern exactly. Multiple sequence alignment was used to compare the amino acid sequences in the *Dof* structural domain of 29 different species of lotus. It was found that the *NnDofs* structural domain sequences were very similar, that they all had the typical CX2CX21CX2C pattern, and that they all had nuclear localization signals (NLS).

Gene duplication is an essential mechanism for generating new evolutionary templates in eukaryotes [46]. In the evolution of angiosperm genomes, genomic duplications have occurred extensively, including whole-genome duplications (WGDs) and segmental duplications [47,48]. Analysis of gene duplications can help us better understand how genes and organisms evolved over time [49]. Ten pairs of segmental duplications and two sets of tandem duplicated *NnDofs* were identified in the lotus genome. Most of the segmental duplicated *NnDofs* had high sequence similarity, while the Ka/Ks of the duplicated gene pairs were less than one. Indicating that all duplicated pairs of *NnDofs* were negatively selected during the evolutionary process, suggesting that to some extent, segmental duplications may be the main amplification mode of the *NnDof* gene family. To some extent, genome duplication may be the main amplification mode of the *NnDof* gene family. In a homozygous analysis of *NnDofs* in lotus and three representative plants, it was found that some *NnDof* genes, such as *NnDof1* and *NnDof10*, were linked to at least three homozygous gene pairs. It is thought that these *NnDofs* may be vital to the generation of the *NnDof* gene family.

### 3.2. Expression Profiling of NnDofs

*NnDofs* exhibit tissue-specific expression patterns, confirming previous research on *Dof* genes in other species. For instance, *NnDof2*, *NnDof5*, *NnDof13*, and *NnDof20* were highly expressed in the seed coat and rhizome (mixed stage), root and mature receptacle, mature stamen, and petiole and leaf, respectively. In addition, except for *NnDof16*, *NnDof19*, and *NnDof28*, all other *NnDofs* showed lower expression levels in the cotyledon. *Dof5.6*/*HCA2* has been reported to positively regulate the formation of interfascicular cambium during vascular tissue development in Arabidopsis [50]. *AtDof2.4*/*AtDof5.8* may be essential in the primary but distinct processes of vasculature formation [51]. Arabidopsis root hair development is ABA-dependent inhibited, attributed to *OBP4*-mediated transcriptional regulation of *RSL2* [52]. Dof genes are definitely widely involved in plant tissue differentiation and development, which explains several *NnDofs*’ differential expression in rhizome (mixed stage), rhizome internode, rhizome elongation zone, and other tissues. In addition, Dof has distinct expression patterns in different tissues and developmental processes in plants. In pepper, *CaDof18* was preferentially expressed in the early stage of flower [14]. Ethylene induces specific increased expression of several *MaDofs* in bananas during fruit ripening [53]. More than half of the A and B1 Dof group members in *Brassica napus* were more highly expressed in the stems and young roots, respectively [54]. *CsDof33* was highly expressed in the terminal buds of tea plants, whereas the expression in young leaves was reversed [55].

Cis-acting elements are essential in gene expression [56], and gene promoter investigation is crucial to understanding the general control of gene expression in plants [57]. In this study, a large number of elements related to light response and meristematic tissue expression were found in the promoter region of *NnDofs*, suggesting that these *NnDofs* may be involved in light signaling pathways or meristematic tissue development. Various hormone responsive elements (ABA, GA, and MeJA response elements) and abiotic stress responsive elements are predicted.

Various investigations into the response of lotus to abiotic stresses have been published. Isolation of bZIP TFs from salt-tolerant lotus root tips enhances the adaptation of transgenic tobacco plants under salt stress [58]. The *NnCIPK6* gene was highly expressed under NaCl treatment in lotus resistant cultivars and was successfully cloned [59]. *NuSTP5*, a monosaccharide transporter family gene, produces stress responses to NaCl, drought, and cold stress [60]. All *NnWRKYs* responded to at least one of SA, JA, and submergence treatments, suggesting that they are extensively involved in abiotic stress [61]. However, studies on the lotus Dof TFs have not been reported yet. According to qRT-PCR analysis, the expression pattern of *NnDofs* was dramatically changed after salt treatment. Under salt stress, the expression of most *NnDofs* was significantly upregulated within 1 h but then progressively decreased after 4 h. It is proposed that several specific *NnDofs* may be engaged in response to salt stress in lotus. Published studies have shown that *Dof* TFs are involved in salt stress resistance of plants through various physiological pathways. Overexpression of *GhDof1* in cotton resulted in a substantial improvement throughout salinity tolerance in wild-type plants, according to prior research [32]. Several *CaDof* genes in pepper were determined as being particularly sensitive to salt stress [14]. The expression of the *ZmDof* gene in maize seedlings was dramatically elevated in those that had been exposed to salt [62]. Salt stress inhibits the transcription of *OsDof15*, which regulates ethylene generation and limits primary root growth in rice by direct contact with the *OsACS1* promoter [34]. *SlDof22* inhibition significantly reduces the expression of the *SlSOS1* gene in tomato, resulting in lower tolerance to salt stress [35]. Under salt conditions, tomato plants that overexpressed the *CDF3* gene maintained growth and boosted yield [22].

## 4. Conclusions

Twenty-nine *NnDofs* were identified in lotus species divided into six subfamilies, the physicochemical properties of which vary and all of which contain conserved zinc finger structures. Segmental duplications may be the primary mode of amplification for the NnDofs gene family. In addition, some *NnDofs* have distinct expression specificities in different tissues and developmental stages. Most of the *NnDofs* were significantly regulated by salt stress treatments. Therefore, *NnDofs* may be engaged in multiple cross-regulatory networks related to lotus development and salt stress responses, and their interactions would help to explain the dynamics of co-regulatory functions of signaling differently in various biological processes. This study provides a foundation for further investigation into the functional properties of the *Dof* gene family in lotus, particularly its role in salt stress resistance.

## 5. Materials and Methods

### 5.1. Identification and Physical Properties Analysis of NnDofs

The lotus genome data used in this study are available under the Nelumbo Genome Database (NGD) [63]. All protein sequences from the lotus genome were scanned by HMMER 3.0 using a hidden Markov model (HMM) of the Dof domain (PF02701), downloaded from the Pfam website (http://pfam.xfam.org/, accessed on 3 May 2021), with an E value of 1 × 10^−5^. The presence of the conserved domain of Dof in the predicted protein was confirmed by NCBI Conserved Domain Database (CDD), Pfam, and SMART. Subcellular locations were predicted using ProtComp 9.0 from Softberry (http://www.softberry.com/, accessed on 3 May 2021). Protein molecular weight (Mw) and theoretical isoelectric point (pI), instability index, aliphatic index, and grand average of hydropathicity (GRAVY) were calculated by Expasy (https://web.expasy.org/protparam/, accessed on 3 May 2021).

### 5.2. Conserved Domain, Conserved Structure, and Phylogenetic Analysis of NnDofs

Multiple protein sequence alignments of Dof domains were constructed using ClustalW, and the results were submitted to GeneDoc software for optimization. After analyzing the full-length sequence, the gene structure pattern distribution of NnDofs was drawn using Tbtools software. The conserved motifs of NnDof sequences in lotus were scanned using MEME (https://meme-suite.org/meme/tools/meme/, accessed on 3 May 2021), with a maximum number of motifs of 10 and the remaining parameters set to default values. The Dof-deduced sequences in Arabidopsis and lotus were aligned using MUSCLE with default settings. Phylogenetic trees were constructed using MEGA 7.0 (http://www.megasoftware.net/, accessed on 5 May 2021) with the neighbor-joining (NJ) method and 1000 bootstraps.

### 5.3. Gene Duplication and Syntenic Analysis of NnDofs

All NnDofs genes were mapped to lotus chromosomes based on physical location information from the database of lotus genome using Circos. The multiple collinear scanning toolkit (MCScanX, http://chibba.pgml.uga.edu/mcscan2/, accessed on 7 May 2021) was used to analyze gene duplication events in lotus, and for Arabidopsis, tomato, soybean, rice, maize, and lotus protein sequences, a collinearity analysis was performed between them. Finally, gene duplication and collinearity were shown using Circos software and Tbtool software with default parameters [64].

### 5.4. Cis-Acting Element Analysis and Protein Interaction Network Prediction of NnDofs

Genomic DNA sequences 2000 bp upstream of each NnDofs transcription start site were extracted and submitted to the PlantCARE database (http://bioinformatics.psb.ugent.be/webtools/plantcare/html/, accessed on 9 May 2021) to predict the cis-acting elements in the NnDofs promoter region.

All putative NnDof protein sequences were uploaded to the webserver STRING (version 11.5; http://string-db.org, accessed on 11 May 2021) to build an interaction network, and the NnDof protein interaction network was mapped using Cytoscape.

### 5.5. Expression Patterns Analysis of NnDofs by RNA-Seq Data

A study by Zhang describes information from transcriptomic data from various tissues of the lotus [63]. To analyze the expression patterns of NnDofs, we generated 20 groups of tissue-specific transcript abundances at different developmental stages based on the data obtained. The 20 groups of tissues are from the seed coat, cotyledon, root, rhizome (mixed), leaf, petiole, receptacle, stamen, petal, carpel, rhizome internode, rhizome elongation zone, and rhizome apical meristem. The seed coat data were taken at 18 days after pollination, and the cotyledon data were taken at 15 days after pollination. Triplicate data were averaged, data were transformed by log_2_^(FPKM+1)^, and heatmaps were created using a plugin within the Tbtools software. All the detailed data are shown in Table S2.

### 5.6. Stress Treatments and Quantitative Real-Time PCR Analysis

*Nelumbo nucifera* cv. ‘Taikonghongqi’, a typical cultivated variety, was used throughout the study. The variety was cultivated in a greenhouse at Fuyang Normal University, Fuyang City, Anhui Province, China, from June 2021 to July 2021. ‘Taikonghongqi’ seeds were surface sterilized in 75% ethanol for 2 min before being peeled open and fully soaked in water. They were then provided an artificial environment with 16 h of light and 8 h of darkness at a temperature of 24 °C for four weeks. Lotus seedlings with similar development status were selected and randomly divided into two groups and cultured under the same culture conditions for 72 h. For the salt-stress treatment group, the roots of the lotus seedlings were exposed to high salt stress (200 mM NaCl) for 24 h. For all treatments, plant materials from three biological replicates were harvested immediately, frozen in liquid nitrogen, and then stored at −80 °C until RNA isolation.

Total RNA was extracted with a plant RNA extraction kit (Huayueyang, Beijing, China) and treated with RNase-free DNase I to remove potential genomic DNA contamination. Qualified RNA was selected as the template to generate first-strand cDNA by gel electrophoresis and A260/A280 ratio determination. Complementary cDNA was generated with reverse transcriptase (TransGen Biotech, Beijing, China). Specific NnDof gene primers were designed using Primer Premier 5, and the expression levels of different sampling cycles were normalized with NnActin gene as a reference gene. Supplementary Table S3 provides all primer information. qRT-PCR was performed using 2×SYBR Green qPCR Mix (with ROX) (Sparkjade, Qingdao, China) and amplified using 96-well plates and a CFX96 TouchTM RT-PCR system (Biorad, Los Angeles, CA, USA). Each reaction was performed in biological triplicate. Data from qRT-PCR amplifications were analyzed using the 2^−ΔΔCt^ method.

## Data Availability

Not applicable.

## Supplementary Materials

The following supporting information can be downloaded at: https://www.mdpi.com/article/10.3390/plants11152057/s1, Table S1: List of the 29 NnDof genes identified in this study; Table S2: RNA-seq data of 29 Nndof genes that were used in this study; Table S3: Primers of NnDof genes used in RT-qPCR; Table S4: The lotus Dof gene sequence identified in this study.
